# Royal Westminster Ophthalmic Hospital

**Published:** 1829-08

**Authors:** 


### HOSPITAL REPORTS,

*(Principally condensed from various Periodical Publications.)*

---

### ROYAL WESTMINSTER OPHTHALMIC HOSPITAL.

*Report of Cases, attended by* Mr. J. Foote, Jun.; *under the Direction of* Mr. Guthrie.

#### *Purulent Ophthalmia of Infants.*

I. Edward Champion, æt. five weeks, was admitted Jan. 8th, 1829. The left eye was observed to be weak the day after he was born, but no discharge was observed until the next day, when it was in great quantity. Three days after, the right was found to be in the same state. The discharge is now thought to be less. Has had advice, and used lotions externally; has taken opening medicines likewise. Its mother is subject to fluor albus.—The Ung. Arg. Nitrat. was applied, after syringing out the eyes with Lotio Aluminis; which was ordered to be used several times a day, on alternate days.

10th.—The discharge is considerably less; the eyes are much better.—Rep. Ung. et Lotio.

13th.—Nearly well. No discharge, except a very slight one in the evening. Opens its eyes with ease.—Rep. Ung. et Lotio.

15th.—Cured.

II. Edwin Carter, æt. eight weeks, admitted Feb. 19th, 1829. The right eye was first affected about three days after birth; the left, a day or two later. It commenced with a great discharge of matter. The lids were much swollen; conjunctivæ of both eyes much injected. Lids still swollen; discharge great; child restless; cornea of the left eye clear, of the right ulcerated and muddy. Has had a leech applied at three different times; blisters to the temples; has taken alteratives and used lotions, without effect. Mother subject to fluor albus.—Appl. Hirudines ij. temp. dextr. Ung. Argent. Nitr. ad sing. ocul. The eyes to be syringed out every hour with the Lotio Aluminis. Ol. Ricini ʒi. secundis horis donec solvetur alvus.

Feb. 24th.—Freely purged by the Ol. Ricini. Can open its eyes itself. Discharge has ceased.—Lotio Aluminis.

27th.—Cured. In attendance on account of a leucoma remaining in the place of the ulcer, which has healed.

III. Edward Shaughslay, admitted March 26th, 1829; ætat. three weeks. The disease began about four days after birth, and has continued nearly three weeks. Great discharge; lids much swollen; child restless. Has had leeches applied twice; lids have been scarified. Mother has fluor albus.

The discharge is rather less; cannot open its eyes itself. On examining the left eye, the cornea appeared muddy, and had a speck on the centre; the conjunctiva much injected. The right eye could not be examined at that time, owing to the patient's resistance. In a few days afterwards it was examined, and appeared very like the left.—Applic. Ung. Argent. Nitr. sing. ocul. Ung. Zinci nocte utend. ad palp. Lotio Aluminis sextis vices utend. in die alternâ. Pulv. alter. nocte sumend. Infus. Sennæ mane.

28th.—Discharge as much as ever; lids less swollen; eyelids more easily opened.—Continue treatment.

31st.—Better. Discharge less.

April 4.—Opens his eyes easily. Discharge less.

9th.—Very little discharge.

11th.—Left nearly well, right much better.—Cont. treatment.

13th.—Left cured; right discharges still a little.

18th.—No discharge. Lotion continued.

21st.—Cured.

IV. Mary Kennard, æt. twelve weeks, admitted March 28th, 1829. Lotio Aluminis alone employed.

When five days old, the left eye became inflamed, and discharged yellow matter the next day. The right was not affected until yesterday. Two leeches were applied near the inner canthus on Wednesday: they bled profusely. Infant is restless at night. Has had castor oil frequently.—Lotio Aluminis sæpe utend.

30.—Much better. Opens her eyes.—Cont. Lotio.

31.—Continues better.—Rep. Lotio. Habeat Pulv. alter.

April 4.—Improving. Rep. Lotio et Pulv.

7.—The discharge more abundant; most from the right eye.—Applic. Ung. Arg. Nitr. ad oculis dextro. Lotio ad ocul. sinist.

10.—The right eye is the best.

21.—Better. Discharge thicker, much the same in quantity.—Rep. Ung. dextro, et Lotio ad sinist. Pulv. alt. rep.

23.—Discharge much less; can open her eyes much better.—Repeat.

27.—Nearly well. Discharge very slight.—Rep.

30.—Discharged, cured.

*Muco-Purulent Ophthalmia. Second Stage of the acute Catarrhal Inflammation.*

V. Jane Hazel, æt. one year, admitted May 7th. Caught cold by sleeping in a room with the windows open. On looking at the child next day, there appeared a black mark, or ecchymosis, circling the lower lid, as if from a blow. This was accompanied by a slightly increased flow of tears. The following night, the lids were much swollen; a secretion, thick and like matter, was discharged. The eye itself had not the appearance of inflammation at that time. The child appeared to suffer much pain, was restless at night.

The present symptoms are, a puriform discharge; lids much swollen; conjunctiva much injected, cornea clear; cannot open the eye; appears to suffer pain, is restless; tongue white and furred; bowels open by medicines. Has used lotions and fomentations to the eye.—The eye was immediately syringed out with some water; then the Unguent. Nigr. was applied. Calomel given to open the bowels. The eye to be kept clean.

8.—Discharge is thicker and in less quantity. Does not appear to suffer so much pain; slept well. Part of the conjunctiva appears to overlap the cornea. Lids are not so much swollen. Bowels freely open. Pulse rather frequent, between eighty and ninety. Tongue looks better; appetite better.—℞. Hydr. Subm. gr. ij.; Pulv. Antim. gr. i. M. fiat pulv. nocte sumend. Lotio Aluminis ter quaterve die utend.

9.—Discharge is less, the eye otherwise much the same; right eye appears to partake in the inflammation. Sleeps tolerably well, but was rather restless. Bowels open, no fever, skin cool, pulse eighty.—Ung. Nigr. sing. ocul. Pulv. rep. Rep. Med. et Lot.

12.—Rather better; swelling of lids less; restless at night. Right much better.—Rep. Ung. ad sing. ocul. Rep. medic.

13.—Much better. Discharge much less; swelling of the lids has subsided; and also of the conjunctiva, it is not so much injected; can open her eye. The right eye is nearly well. Bowels open, skin cool. The powder does not remain upon her stomach. —Rep. Lotio. ℞. Hyd. Subm. gr. iij.; P. Antim. gr. iss. nocte sumend. Rep. med.

14.—Much better. Rep. Ung. et med.

17.—Has not attended for three days. The disease has returned nearly as bad as ever. The child is very restless; feverish at night; pulse ninety; no appetite; urine high coloured; can open its eye but little.—The eye was syringed out, and the Ung. Nigr. applied. Hirudines iij. temp. sinist. statim applic. Pulv. alter. nocte et mane. Inf. Sennæ mane.

18.—Discharge less; slept badly; bowels open.—Lotio Alum. quater die. Rep. medic.

20.—Slept better the last two nights, and is altogether better. —Rep. omnia. 21.—Rep. Ung. et Pulv.

22d.—Better. Rep. Pulv.

25.—Discharge very slight; nearly well.—Rep. Ung. et Pulv.

27.—Cured.

*Sloughing Ulcer of the Cornea.*

VI. Mary Mills, æt. ten, admitted Dec. 27, 1828. A large deep ulcer on the cornea, at its under and outer edge. The upper part of the ulcer sloughs; the rest of the cornea clear. Iris quite dilatable, but changed in colour, and red vessels are running upon it. Has great pain in the eye. Is restless at night; almost delirious.—Applic. Argent. Nitrat. in substance. Five ounces of blood taken by cupping. Pulv. Jalapæ comp. ʒss. mane.

28.—Says she is much better, that the touching the eye relieved her; slept well; has no pain.—Rep. Pulv.

30.—Says she is going on well.—Rep. Pulv.

31.—Repeat.

January 8.—Ulcer is spreading.—Appl. Argent. Nitrat. Pulv. repet.

13.—The ulcer is getting white towards the centre, showing a disposition to spread.—Arg. Nitr. Pulv. rep.

15.—Ulcer filling up.

20.—Appl. Arg. Nitr. Pulv. 22.—Better.

27.—Cured. Iris has perfectly regained its colour. A large leucoma is left.

*Chronic Catarrhal Inflammation of Conjunctivæ.*

VII. John Loughlin, æt. three years and a half, admitted Jan. 1, 1829. His eyes have been affected for three months, owing to frequent exposure to cold. There is much lacrymation; lids swollen and discoloured; complains of pain. The eyes themselves could not be examined, owing to the child's resistance. Bowels regular.—Ung. Arg. Nitr. sing. ocul. Inf. Sennæ mane.

6.—A little better. Continue.

10.—Much better; eyes discharge greatly. Rep.

15.—Worse. Rep. med.

20.—Much worse. In consequence, the stimulant plan was changed for the antiphlogistic: Hirudines iij. sing. ocul. Hydr. Subm. gr. iv. omni nocte. Inf. Sennæ mane sum.

22.—Can open his eyes: is much better.—Hirud. i. sing. ocul. Pulv. rep. et Inf. Sennæ.

25.—Rep. med.

27.—Much better.—Hirud. ij. sing. ocul. Pulv. rep. et Inf.

31.—Hirud. i. sing. ocul. Rep. med.

Feb. 5.—Hirud. i. sing. ocul. Rep. med.

7.—Rep. med.

10.—Nearly cured. Rep. med. 14th.—Cured.

*Iritis of the left Eye.*

VIII. John Perkins, æt. thirty-three, admitted June 13th, 1829. The eye has been affected for a fortnight: in the commencement it was attended with deep-seated pain in the eye, and round the brow, which became much worse in the course of a few days; great dimness of sight, greater in the morning. There is not much pain now, nor does the light affect him much. About eight months ago had primary sores on the penis, for which he took mercury so as to affect the system; and for the last five months has had pains in his shin-bones, with sore throat, &c. Has merely applied leeches and taken aperients. The iris slightly dilates, is rather changed in colour. Zone of pink vessels surrounding the cornea. —℞. Hyd. Subm. gr. ij. quartis horis. Appl. gutt. Belladonnæ.

14.—The eye feels easy; can see better; mouth not sore; pupil regular; iris has nearly regained its colour. Had a violent pain round the brow and in the forehead last night, and on rising this morning.—Cucur. cruent. ad. f℥x. temp. sinist. Pil. Cal. gr. iij.; Pulv. Opii gr. $\frac{1}{4}$, sextis horis sumend.

On the 23d of the same month, he sent a person to return thanks, stating that his eye is perfectly recovered, that he feels no pain, and can see as well as ever.

